# Days out of role and somatic, anxious-depressive, hypo-manic, and psychotic-like symptom dimensions in a community sample of young adults

**DOI:** 10.1038/s41398-021-01390-y

**Published:** 2021-05-13

**Authors:** Jacob J. Crouse, Nicholas Ho, Jan Scott, Nicholas G. Martin, Baptiste Couvy-Duchesne, Daniel F. Hermens, Richard Parker, Nathan A. Gillespie, Sarah E. Medland, Ian B. Hickie

**Affiliations:** 1 Youth Mental Health & Technology Team, Brain and Mind Centre, University of Sydney, Sydney, Australia; 2 Academic Psychiatry, Institute of Neuroscience, Newcastle University, Newcastle, UK; 3 Diderot University, Paris, France; 4 Norwegian University of Science and Technology, Trondheim, Norway; 5 QIMR Berghofer Institute of Medical Research, Brisbane, Australia; 6 Institute for Molecular Bioscience, The University of Queensland, Brisbane, Australia; 7 ARAMIS Laboratory, Paris Brain Institute, Paris, France; 8 Thompson Institute, University of the Sunshine Coast, Birtinya, Australia; 9 Virginia Institute for Psychiatric and Behavioral Genetics, Virginia Commonwealth University, Virginia, USA

**Keywords:** Depression, Bipolar disorder, Schizophrenia

## Abstract

Improving our understanding of the causes of functional impairment in young people is a major global challenge. Here, we investigated the relationships between self-reported days out of role and the total quantity and different patterns of self-reported somatic, anxious-depressive, psychotic-like, and hypomanic symptoms in a community-based cohort of young adults. We examined self-ratings of 23 symptoms ranging across the four dimensions and days out of role in >1900 young adult twins and non-twin siblings participating in the “19Up” wave of the Brisbane Longitudinal Twin Study. Adjusted prevalence ratios (APR) and 95% confidence intervals (95% CI) quantified associations between impairment and different symptom patterns. Three individual symptoms showed significant associations with days out of role, with the largest association for impaired concentration. When impairment was assessed according to each symptom dimension, there was a clear stepwise relationship between the total number of somatic symptoms and the likelihood of impairment, while individuals reporting ≥4 anxious-depressive symptoms or five hypomanic symptoms had greater likelihood of reporting days out of role. Furthermore, there was a stepwise relationship between the total number of undifferentiated symptoms and the likelihood of reporting days out of role. There was some suggestion of differences in the magnitude and significance of associations when the cohort was stratified according to sex, but not for age or twin status. Our findings reinforce the development of early intervention mental health frameworks and, if confirmed, support the need to consider interventions for subthreshold and/or undifferentiated syndromes for reducing disability among young people.

## Introduction

The increasing number of young adults who are not in employment, education, and training is a major global challenge^1^. After adjusting for geopolitical variations, it is clear that depression, bipolar disorder, and schizophrenia are leading contributors to all types of functional impairment and social disengagement in young people^2^. As such, understanding the inter-relationships between clinical and functional phenomena and increasing efforts to prevent and/or modify the onset and course of mental disorders and impairment are needed^3,4^. Recent research demonstrates that young people who experience mental health problems and accompanying impairment often present with conditions that do not meet diagnostic criteria for a full-threshold episode of a mental disorder, but with so-called “attenuated” or “subthreshold” syndromes or with undifferentiated symptoms that impair day to day functioning^5–7^. Despite this, there are few studies that examine the relationships between impairment and these broader dimensions of psychopathology. Given the emphasis on public health programs and early intervention services that target young people in the early stages of mental illness, it is important to clarify whether and how individual symptoms, different symptom patterns or dimensions, and/or total symptom load are linked with functioning.

Clinical and community studies over the last 30 years have provided support for dimensional models of psychopathology, and the notion that the magnitude of functional impairment is often associated with incremental increases in symptom load or burden. Most studies to date have focused on psychological symptoms (such as anxious-depressive, hypomanic, or psychotic-like experiences) or on somatic (physical) symptoms. For example, a study of adults in the Epidemiological Catchment Area study showed that people experiencing minor depression with mood disturbance (based on the Diagnostic Interview Schedule) had a 1.6-fold greater risk of self-reported disability days compared to asymptomatic individuals, while those with major depression had a 4.8-fold greater risk of disability days^8^. Further, given its higher relative prevalence, minor depression was associated with 51% more days out of role than major depression^8^. Likewise, self-reported somatic symptoms such as sleep-wake disturbances are common in general population samples and are associated with high rates of self-reported disability^9,10^. For example, a primary care-based study of 15 common physical symptoms in adults (e.g., fatigue, joint or limb pain, and headache) reported that all self-reported symptoms were significantly associated with self-reported impairment, and that symptom load (the total number of symptoms) was strongly associated with functional status^11^. Psychotic experiences and attenuated psychotic syndromes have received increased attention in recent years following recognition of their higher-than-expected prevalence in the community and relationships with impairment. For example, two partially overlapping studies of high school and university students demonstrated that several subtypes of self-reported psychotic-like experiences (bizarre experiences, perceptual abnormalities, persecutory ideas, and grandiosity) were each associated with impaired self-reported functioning^12,13^, and clinical studies of non-psychotic help-seeking young people accessing youth mental health services have reported similar relationships between self- and clinician-rated impairment and self-reported bizarre experiences and persecutory ideas^14^. Only a handful of studies have examined relationships between impairment and hypo-manic symptoms and syndromes. A re-analysis of five sites of the Epidemiological Catchment Area study showed that Diagnostic Interview Schedule ascertained “subthreshold manic/hypomanic symptoms” (which together represent a similar condition to brief hypomania meeting the Zurich criteria) were associated with increased need for welfare/disability benefits^15^, and two studies of community-based cohorts of young people showed that individuals with clinically-determined subthreshold bipolar disorder in adolescence had greater self-rated and clinician-rated functional impairment in early adulthood compared to asymptomatic individuals^16,17^. Altogether, these and other studies demonstrate incremental relationships between a continuum of psychopathology and the level of impairment^18–23^.

An important limitation of previous studies has been their focus on single symptom dimensions or syndromes (i.e., they do not explore a broad range of somatic and psychological symptoms in conjunction). Recently, we showed that subthreshold presentations of depression-like, hypomanic-like, and psychotic-like syndromes were each associated with greater level of perceived impairment in young adults compared to those with no subthreshold syndrome and that experiencing >1 subthreshold syndrome was associated with even greater impairment^24^. As many young people experience non-specific admixtures of symptoms in the early stages of mental illness^25^, and comorbidity is the rule rather than the exception in young people^26^, there is a need to examine broader relationships of impairment and patterns of symptoms occurring both within specific dimensions and across multiple dimensions^27,28^.

Accordingly, the goal of this study is to model the associations between impairment and levels of symptoms within and across somatic, anxious-depressive, hypomanic, and psychotic-like dimensions in a large community-based cohort of twins and non-twin siblings (*Brisbane Longitudinal Twin Study*). Here, we selected “days out of role” as a measure of impairment because of its wide use (e.g., WHO World Mental Health surveys), convenience, objectivity, and established links with mental health across a range of cultures, countries, and clinical and non-clinical settings^6,8,29–33^. Specifically, the current study examines the relationships between self-rated days out of role and:

1. Individual self-reported symptoms (i.e., somatic, anxious-depressive, hypomanic, and/or psychotic-like experiences, irrespective of symptom dimension).
2. Total number of self-reported symptoms within each dimension.
3. Total number of undifferentiated symptoms (i.e., across the four dimensions).

## Subjects and methods

### Ethical approval and observational study reporting

Ethical approval was obtained from the Human Research Ethics Committee at the Queensland Institute of Medical Research for all Brisbane Longitudinal Twin Study research projects (reference numbers: EC00278, P1212). This study follows the Strengthening the Reporting of Observational Studies in Epidemiology (STROBE) guidelines^34^ (see STROBE checklist in Supplementary Materials). Written, informed consent was obtained from participants, and their parents if applicable (i.e., participants aged under 18).

### Study participants

The Brisbane Longitudinal Twin Study (BLTS) is an ongoing prospective cohort study of twins and their siblings in Queensland, Australia, conducted at the Queensland Institute of Medical Research (QIMR). The BLTS began in 1992, recruiting twins around age 12 from primary and secondary schools in the greater Brisbane area in Queensland via media appeals, word of mouth, or from the Australian Twin Registry^35,36^. The BLTS was originally conceived to study the development of melanocytic naevi (moles) and cognition during adolescence, but additionally includes a rich variety of biological, psychological, and behavioral assessments of personality, substance use, neurobiology, acne, and taste and olfaction, among others^35,37–39^. As described elsewhere^35,38^, the BLTS cohort appears representative regarding psychometric IQ, the proportions of twins by sex and zygosity are in keeping with population expectations, and the ethnic distribution reflects the population structure of Queensland at the time of recruitment (mostly European ancestry and minorities of Asian ancestry). All BLTS cohort members have been invited to participate in re-assessments around age 14, 16, 19, and most recently, 25^40^. The current study focuses on the “Nineteen and Up” (19Up) study, for which data were collected between 2009–2016^37^ (a flow diagram is available in Supplementary Fig 1).

### Eligibility criteria

Prospective recruits were ineligible for initial entry into the BLTS (i.e., around age 12) if they had: (1) vision/hearing impairments; (2) history of closed head injury; (3) or significant mental or neurological disorders. For this study, participants were eligible if they responded to self-report questionnaires of mental health symptoms and functioning in the “19Up” wave. For participants who completed questionnaires on multiple occasions in 19Up, we selected their most recent responses. There were no age restrictions set for inclusion in the current study.

### Assessments

#### Sociodemographic characteristics

Data regarding age, sex, zygosity, marital status, occupation, and highest level of education were collected using self-report questionnaires.

#### Self-rated symptoms

**Fig. 1:**
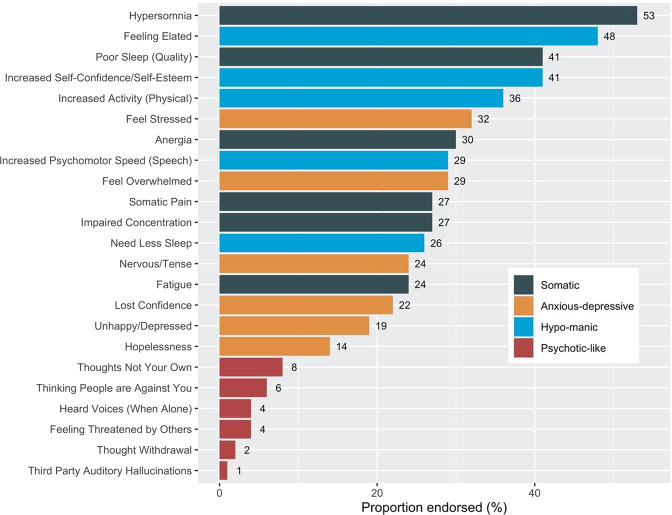
Prevalence of self-rated somatic, anxious-depressive, hypomanic, and psychotic-like symptoms in a community-based cohort of young adults. Percentages are reported to the nearest whole number.

- Somatic and anxious-depressive symptoms: Measured using the 6-item SOMA and 6-item PSYCH subscales of the 12-item Somatic and Psychological Health Report (SPHERE-12)^32^. The SOMA-6 subscale includes a subset of six items from the Schedule of Fatigue and Anergia (SOFA)^41^, which were initially included based on their ability to predict a SOFA case^32^. The PSYCH-6 subscale includes a subset of six items from the General Health Questionnaire (GHQ)^42^, initially included based on their being from the depression/anxiety domain and their ability to predict a mental disorder on the GHQ^32^. The SPHERE-12 subscales have high internal consistency (Cronbach’s α: PSYCH-6 = 0.90; SOMA-6 = 0.80) and high test-retest reliability over two occasions 3–6 months apart in a general practice sample (intraclass correlation: PSYCH-6 = 0.81; SOMA-6 = 0.80)^32^. For each symptom item, participants were required to answer the question “Over the past few weeks have you been troubled by…” with one of three responses: “never or some of the time”, “a good part of the time”, or “most of the time”. We coded these symptoms as present (1) (“a good part of the time” or “most of the time”) or absent (0) (“never or some of the time”). The items are shown in Fig. 1 and a full list of questions is in Supplementary Table 1.
- Hypomanic symptoms: Measured by five items partially derived from symptoms included in the DSM-IV, bipolar at-risk criteria^43,44^, and Altman Self-Rating Mania Scale^45^. For each item, participants were required to answer yes/no to the question “Have you ever experienced a definite period where for more than 2 or 3 days you…”. These items are useful for assessing hypomanic symptoms/syndromes or bipolar spectrum conditions in young people^46^, which are more prevalent in the general population than previously thought^47,48^. These items are shown in Fig. 1 and a full list of questions is in Supplementary Table 1.
- Psychotic-like symptoms: Measured by six items of positive psychotic experiences that are prevalent in clinical and community-based samples of young people and related to impairment^12–14^. For each item, participants were required to answer yes/no to the question: “Have you ever…”. The six items are shown in Fig. 1 and a full list of item questions is in Supplementary Table 1.

#### Self-rated days out of role

Days out of role was measured using a modified version of the World Health Organization’s Disability Assessment Schedule (WHO-DAS)^49^. Participants were asked: “During the last few weeks how many days in total were you unable to carry out your usual daily activities fully?” Previous work has reported good concordance with a similarly worded question and payroll records of employed people^50^. Here, participants were classified as having any days out of role if they responded with ≥1.

### Statistical analyses

Analyses were conducted using R (version 4.02) with the RStudio IDE^51^ and run on macOS Catalina (version 10.15.7). Continuous measures are reported as means and standard deviations and binary measures as numbers and percentages. Complete case analysis was used. Our main analyses included three models: (1) absence/presence of 23 individual symptoms and age, sex, and twin status; (2) total number of symptoms within four domains (anxious-depressive, somatic, hypomanic, and psychotic-like) and age, sex, and twin status; (3) total number of “undifferentiated” symptoms and age, sex, and twin status. In exploratory analyses, we re-ran each model separately in males and females. We calculated adjusted prevalence ratios (APRs) as a measure of association between the predictor and outcome variables. Adjusted prevalence ratios have been reported to be preferred over odds ratios for clustered, cross-sectional epidemiological (e.g., twins and siblings) and samples with common outcomes (e.g., >10%)^52^. Using the “prLogistic” package (version 1.2), we estimated APRs using random effects logistic regression models and estimated 95% confidence intervals (95% CI) using the “delta” method, which produces an approximate standard error for the APR to estimate the 95% CI^52,53^. To control for multiple comparisons in our main analyses, we report false discovery rate (FDR) *P*-values alongside raw *P*-values, calculated using the Benjamini–Hochberg procedure^54^. We reserve use of “significant” for coefficients with an FDR-adjusted *P*-value below <0.05. Analyses were conducted by J.J.C. and N.H. between May and September 2020.

## Results

### Sample characteristics

Of 2773 individuals who participated in the “19Up” wave of the BLTS^37^, a total of 1904 individuals met eligibility criteria for this study, and sociodemographic characteristics are in Table 1. Of these 1904 participants, 791 were male and 1113 were female. The median age was 26 (IQR = 23–29). There were 605 monozygotic twin individuals, 753 dizygotic twin individuals, and 545 non-twin siblings (zygosity was undetermined for one individual).

**Table 1:** Sociodemographic characteristics of sample (*N* = 1904).

	*N* (%) or *M* (SD)
Age, years	26.3 (3.9)
Sex (female)	1113 (58.5%)
*Marital status*^a^	
Married	466 (24.5)
Separated, divorced, and widowed	41 (2.2)
Never married	1395 (73.3)
*Primary occupation*^a^	
Full-time	1184 (62.3)
Part-time	238 (12.5)
Studying	252 (13.2)
Home duties	106 (5.6)
Employed, not working (e.g., illness)	28 (1.5)
Receiving sickness/disability benefits	16 (0.8)
Volunteer	10 (0.5)
Unemployed	62 (3.3)
Prefer not to answer/don’t know	6 (0.3)
*Education (highest level)*^a^	
Postgraduate degree	275 (14.5)
Undergraduate degree	840 (44.2)
Certificate/diploma	484 (25.4)
Junior/senior high school	299 (15.7)
No formal education	1 (0.1)
Prefer not to answer/don’t know	3 (0.2)

^a^Missing for *N* = 2.

The sample self-reported a cumulative total of 2310 days out of role in the few weeks prior to assessment. Over one-quarter reported at least one day out of role (28%, 540/1904). Females were more likely to report one or more days out of role than males (32.2% vs. 23.0%) (*χ*^2^ = 18.63, df = 1, *P* < 0.001). The prevalence of self-rated somatic, anxious-depressive, psychotic-like, and hypomanic symptoms ranged from 1 to 53% (Fig. 1). The five most prevalent symptoms were “hypersomnia” (53%), “feeling elated” (48%), “increased self-confidence/self-esteem” (41%), “poor sleep (quality)” (41%), and “increased activity (physical)” (36%). The five least prevalent were all psychotic-like symptoms. Participants endorsed between 0–22 symptoms, with a median of five symptoms (see Supplementary Table 2 for the proportions of participants endorsing 0–22 symptoms).

### Days out of role and individual symptoms

Our first set of models examined the relationships between days out of role and the 23 individual symptom items (see Supplementary Table 3). In the total sample, participants had a significantly increased likelihood of having days out of role if they endorsed impaired concentration (APR = 1.59), increased psychomotor speed (speech) (APR = 1.40), and fatigue (APR = 1.34). Hypersomnia (APR = 1.27) and hopelessness (APR = 1.36) were no longer significantly associated with having days out of role after adjusting for multiple comparisons.

### Days out of role and total number of somatic, anxious-depressive, hypomanic, and psychotic-like symptoms

**Fig. 2:**
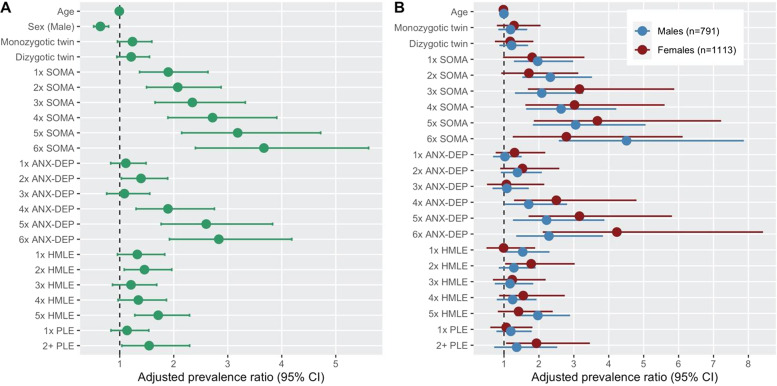
Relationships between days out of role and the number of somatic, anxious-depressive, hypo-manic, and psychotic-like symptoms in a community-based cohort of young adults. **A** Model for total sample; **B** separate models stratified by sex. SOMA somatic symptoms, ANX-DEP = anxious-depressive symptoms, HMLE hypomanic, PLE psychotic-like symptoms.

Our second set of models examined the relationships between days out of role and the number of symptoms in each dimension (Note: the psychotic-like symptom dimension was truncated at ≥2 symptoms due to the distribution of self-ratings; see Supplementary Table 4). In the total sample (Fig. 2A and Table 2), there were significant stepwise relationships between likelihood of having days out of role and all levels of the somatic dimension: one somatic symptom (APR = 1.90), two somatic symptoms (APR = 2.07), three somatic symptoms (APR = 2.34), four somatic symptoms (APR = 2.72), five somatic symptoms (APR = 3.18), and six somatic symptoms (APR = 3.67). The patterns of association for days out of role and anxious-depressive symptoms was somewhat curvilinear: two anxious-depressive symptoms (APR = 1.39) (however this was no longer significant when adjusting for multiple comparisons), four anxious-depressive symptoms (APR = 1.89), five anxious-depressive symptoms (APR = 2.60), and six anxious-depressive symptoms (APR = 2.83). Finally, endorsing ≥2 psychotic-like symptoms was not significantly associated with having days out of role after adjusting for multiple comparisons (APR = 1.54), while endorsing five hypomanic symptoms (APR = 1.71) was associated with having days out of role. As with the individual symptoms model, there was some suggestion of sex differences (Fig. 2B).

**Table 2:** Relationships between somatic, anxious-depressive hypo-manic, and psychotic-like, symptoms and “days out of role” in a community sample of young adults.

Symptom dimension	APR Whole sample^a,b,c^	Raw *p*-value	FDR-adjusted *p*-value
*Somatic*			
0	1.00		
1	1.90 (1.37–2.63)	***	***
2	2.07 (1.50–2.88)	***	***
3	2.34 (1.65–3.32)	***	***
4	2.72 (1.89–3.91)	***	***
5	3.18 (2.15–4.72)	***	***
6	3.67 (2.40–5.61)	***	***
*Anxious-depressive*			
0	1.00		
1	1.11 (0.83–1.49)	NS	NS
2	1.39 (1.03–1.89)	*	NS
3	1.09 (0.76–1.56)	NS	NS
4	1.89 (1.30–2.75)	***	**
5	2.60 (1.77–3.83)	***	***
6	2.83 (1.92–4.19)	***	***
*Hypomanic*			
0	1.00		
1	1.32 (0.96–1.83)	NS	NS
2	1.46 (1.08–1.96)	*	*
3	1.21 (0.87–1.68)	NS	NS
4	1.34 (0.97–1.86)	NS	NS
5	1.71 (1.28–2.29)	***	***
*Psychotic-like*			
0	1.00		
1	1.13 (0.84–1.54)	NS	NS
2+	1.54 (1.04–2.29)	*	NS
Age	0.99 (0.97–1.02)	NS	NS
*Sex*			
Female	1.00		
Male	0.64 (0.52–0.79)	***	***
*Twin status*			
Not a twin	1.00		
Monozygotic	1.24 (0.96–1.59)	NS	NS
Dizygotic	1.21 (0.94–1.55)	NS	NS

APR adjusted prevalence ratio, *NS* non-significant (*p* > 0.05).

*p < 0.05.

**p < 0.01.

***p < 0.001.

^a^Adjusted for age.

^b^Adjusted for zygosity.

^c^Adjusted for sex.

### Days out of role and total number of undifferentiated symptoms

**Fig. 3:**
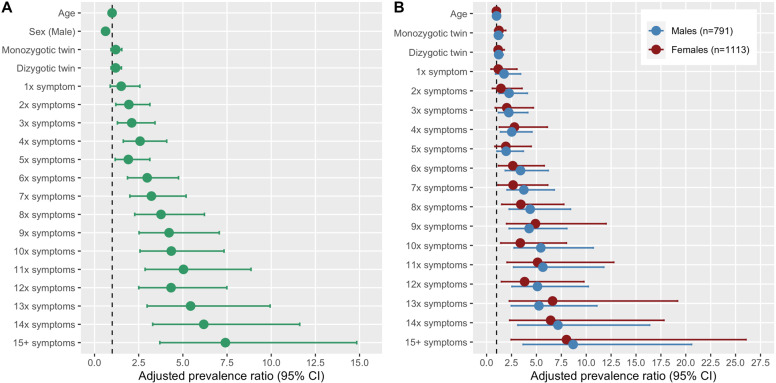
Relationships between days out of role and the total number of undifferentiated symptoms in a community-based cohort of young adults. **A** Model for total sample. **B** Separate models stratified by sex.

Our third and final set of models examined the relationships between days out of role and the total number of undifferentiated symptoms across the four dimensions (Note: the total number of symptoms was truncated at ≥15 symptoms due to the distribution of symptom ratings; see Supplementary Table 2). About 85% of the sample endorsed at least one symptom. In the total sample (Fig. 3A and Table 3), there was a linear relationship between having any days out of role and endorsing ≥2 symptoms, for example: two symptoms (APR = 1.94), 6 symptoms (APR = 2.98), 10 symptoms (APR = 4.34), 14 symptoms (APR = 6.18), and 15+ symptoms (APR = 7.41). Again, there was some suggestion of sex differences (Fig. 3B).

**Table 3:** Relationships between number of “undifferentiated” symptoms and “days out of role” in a community sample of young adults.

	APR whole sample^a,^^b,^^c^	Raw *p*-value	FDR-adjusted *p*-value
*Number of symptoms*			
0	1.00		
1	1.51 (0.89–2.57)	NS	NS
2	1.94 (1.20–3.13)	**	*
3	2.11 (1.30–3.42)	**	**
4	2.58 (1.63–4.08)	***	***
5	1.91 (1.17–3.13)	**	*
6	2.98 (1.87–4.75)	***	***
7	3.22 (2.00–5.18)	***	***
8	3.76 (2.27–6.22)	***	***
9	4.22 (2.52–7.06)	***	***
10	4.34 (2.58–7.33)	***	***
11	5.03 (2.86–8.86)	***	***
12	4.33 (2.50–7.49)	***	***
13	5.44 (2.98–9.94)	***	***
14	6.18 (3.29–11.61)	***	***
15+	7.41 (3.70–14.84)	***	***
*Age*	0.99 (0.97–1.02)	NS	NS
*Sex*			
Female	1.00		
Male	0.62 (0.50–0.77)	***	***
*Twin status*			
Not a twin	1.00		
Monozygotic	1.20 (0.93–1.54)	NS	NS
Dizygotic	1.19 (0.93–1.52)	NS	NS

*APR* adjusted prevalence ratio, *NS* non-significant (*p* > 0.05).

*p < 0.05

**p < 0.01

***p < 0.001

^a^Adjusted for age.

^b^Adjusted for zygosity.

^c^Adjusted for sex.

## Discussion

In this community-based cohort of young adults, we identified both general and specific relationships between the likelihood of having ≥1 day out of role and experiencing symptoms associated with mental ill-health. Before interpreting our findings, it is worth qualifying first that symptoms and impairment were self-rated in this study, and these relationships may not be equivalent to those that would be observed if symptoms and impairment were rated by trained clinicians or other observers; however, many previous studies in this area relied similarly on self-ratings^9–13^. Nonetheless, we focus this discussion on three key observations. First, specific symptoms including hypersomnia, impaired concentration, fatigue, impaired sleep quality, and increased psychomotor speed (speech) were each associated with having days out of role, with more consistent associations noted for hypersomnia and impaired concentration (i.e., significant in the total sample and one of the sex-stratified models). Second, somatic symptoms were a strong predictor of having days out of role. Specifically, endorsing at least one somatic symptom was associated with elevated likelihood of having days out of role, and there was a stepwise relationship between an increasing number of somatic symptoms and the likelihood of having days out of role. Finally, there was a clear stepwise relationship between total undifferentiated symptom burden (including somatic, anxious-depressive, hypomanic, and psychotic-like experiences, irrespective of symptom dimension) and the likelihood of having any days out of role.

To begin with, our finding that specific somatic symptoms (e.g., hypersomnia) and the total number of somatic symptoms were predictors of impairment has high face validity, and adds to the growing body evidence from clinical and community-based studies demonstrating a strong and robust link between impairment and somatic symptoms and syndromes across the lifespan^11,32,55–58^. The likelihood of having days out of role increased linearly with endorsement of one to six somatic symptoms (relative to none) and the APRs between impairment, and the total number of somatic symptoms was consistently higher compared to an equivalent number of symptoms from the other dimensions. For example, while there was a significant relationship between impairment and reporting ≥4 anxious-depressive symptoms, the likelihood of impairment was about the same for 4 anxious-depressive symptoms (APR = 1.89) as a single somatic symptom (APR = 1.90). Similarly, we found that participants had to endorse all five hypo-manic symptoms before likelihood of impairment was statistically significant. While some studies have reported associations between hypomanic symptoms and impairment^15^, other studies of bipolar disorders or transdiagnostic samples tend to show stronger relative associations with impairment for depressive compared to small numbers of hypo-manic symptoms^59–61^. Finally, consistent with clinical and community-based studies, participants endorsing psychotic-like symptoms had elevated likelihood of impairment^62,63^, however, this finding was not statistically significant when adjusting for multiple comparisons. These associations regarding the number of hypomanic and psychotic-like symptoms mirror the findings of our previous study in this sample, in which we reported significant associations between the perceived level of functional impairment associated with self-reported mental health problems and the presence of a hypomanic or psychotic-like subthreshold syndrome (operationalised as ≥5 hypomanic or ≥2 psychotic-like symptoms, respectively)^24^. Altogether, we interpret these findings to indicate that a lower threshold of somatic symptoms is sufficient to precipitate impaired role functioning (if we assume a causal relationship), while crossing a higher threshold for anxious-depressive, hypomanic, and psychotic-like symptoms is necessary to produce impairment.

### Limitations

Our findings should be interpreted in the context of several limitations. First, participants self-reported their symptoms and impairment, and we did not model the likely impacts of severity and persistence of symptoms on impairment^58^. Second, somatic and anxious-depressive symptoms were measured “over the past few weeks”, whereas hypo-manic and psychotic-like symptoms were lifetime questions (“Have you ever…”); these differences may partly account for the lower relative associations between hypomanic and psychotic-like symptoms. Third, we cannot conclude whether somatic symptoms were medically explained or not, which has implications for their management^64,65^. Fourth, while we adjusted our models for twin status and calculated APRs as a recommended measure of association for cross-sectional, clustered data^52^, we treated our study participants as singletons and interpret the findings at the individual level rather than the family or twin-pair level. Fifth, this study was cross-sectional, and we cannot truly assume the direction of causality of any association.

### Potential implications

There are several potential implications of this study. First, somatic symptoms were common (endorsed by 24–53% of the sample) and the stepwise relationship between the total number of somatic symptoms, and the likelihood of impairment suggests that reducing specific symptoms or the total symptom load could help alleviate impairment. Like their older adult counterparts, young adults with impairing somatic symptoms may benefit from psychological therapies such as cognitive behavior therapy^66^. Speculatively, lifestyle modifications with potentially “broad spectrum” benefits, such as increasing exercise or improving sleep-wake cycles, might improve specific (e.g., hypersomnia, impaired concentration) and/or overall somatic symptoms^67–69^. Second, our finding that the total number of undifferentiated symptoms (irrespective of dimension) showed a strong linear association with impairment is a novel finding in this type of sample. As above, it is possible that encouraging lifestyle modifications that are have been shown to improve a range of mental health symptoms, such as improving sleep disturbance and/or sleep-wake cycles^70–72^, could reduce the overall burden of symptoms and partially alleviate associated impairment. Importantly, these types of lifestyle modifications and related interventions are scalable, cost-effective, and engagement can be encouraged via public health programs or increasingly popular digital technologies (e.g., wearable activity/sleep monitors)^73,74^.

Altogether, we find evidence for general and specific relationships between self-rated mental health symptoms and self-reported impairment and show that somatic symptoms are associated with impairment to a greater degree than anxious-depressive, hypomanic, and psychotic-like symptoms in a community-based young adult sample. Health professionals should be alert to the increased risk of functional impairment in young adults presenting with somatic symptoms.

## Supplementary information

Supplementary Figure

Supplementary Tables

## Data Availability

The data that support the findings of this study were made available to authors via the BLTS research committee (that approved the cohort study). The authors confirm that the summary data for all variables supporting the findings of this study are included within the article and its supplementary materials. The raw data are being used at the lead research centers and form part of an ongoing program of research and data are only made available upon written request to the BLTS research committee. Data are not publicly available due to confidentiality restrictions and because research participants did not give permission for dissemination beyond the BLTS research team.
