# Supplementary material for: Days out of role and somatic, anxious-depressive, hypo-manic, and psychotic-like symptom dimensions in a community sample of young adults
Source: Transl Psychiatry. 2021 May 13;11:285. doi: 10.1038/s41398-021-01390-y (PMC8119948; doi:10.1038/s41398-021-01390-y)
Supplement: Supplementary file 1 — Supplementary Figure [file 41398_2021_1390_MOESM1_ESM.docx]

**Supplementary Figure 1. Participant flow-diagram of the ‘19Up’ wave of the Brisbane Longitudinal Twin Study. (**Adapted from 19Up cohort paper ^1^)


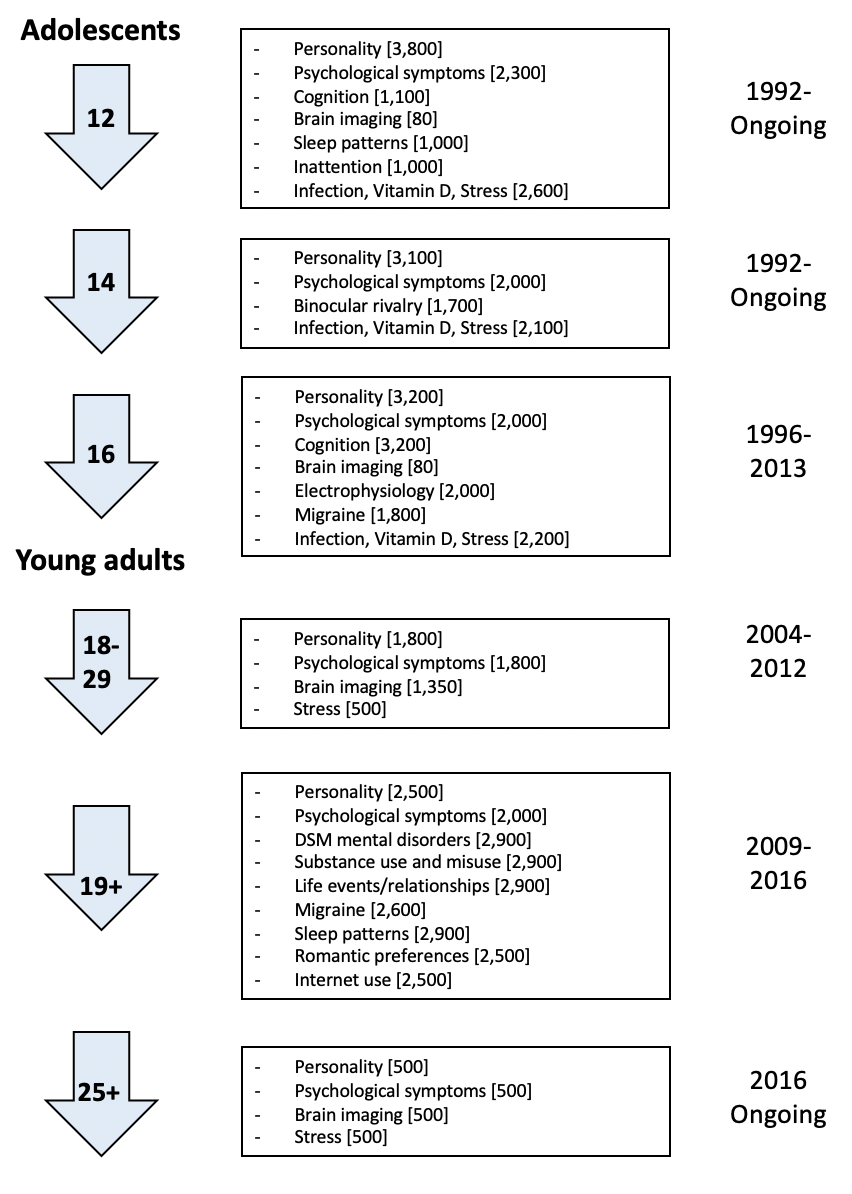


**REFERENCES**

1 Couvy-Duchesne, B. *et al.* Nineteen and Up study (19Up): understanding pathways to mental health disorders in young Australian twins. *BMJ open* **8**, e018959, doi:10.1136/bmjopen-2017-018959 (2018).
