# Supplementary material for: Days out of role and somatic, anxious-depressive, hypo-manic, and psychotic-like symptom dimensions in a community sample of young adults
Source: Transl Psychiatry. 2021 May 13;11:285. doi: 10.1038/s41398-021-01390-y (PMC8119948; doi:10.1038/s41398-021-01390-y)
Supplement: Supplementary file 2 — Supplementary Tables [file 41398_2021_1390_MOESM2_ESM.docx]

**Supplementary Table 1. Item short names and item questions for anxious-depressive, somatic, psychotic-like, and hypo-manic like symptoms.**

|  | **Item short name** | **Item question** |
| --- | --- | --- |
|  |  | *Over the past few weeks have you been troubled by:* |
| **Anxious-Depressive** | Feel Nervous or Tense | Feeling nervous or tense |
|  | Feel Unhappy/Depressed | Feeling unhappy and depressed |
|  | Feel Stressed | Feeling constantly under strain |
|  | Feel Overwhelmed | Everything getting on top of you |
|  | Lost Confidence | Losing confidence |
|  | Hopelessness | Being unable to overcome difficulties |
| **Somatic** | Somatic Pain | Muscle pain after activity |
|  | Hypersomnia | Needing to sleep longer |
|  | Fatigue | Prolonged tiredness after activity |
|  | Impaired Sleep (Quality) | Poor sleep |
|  | Impaired Concentration | Poor concentration |
|  | Anergia | Tired muscles after activity |
|  | | |
|  |  | *Have you ever...* |
| **Psychotic-like** | Thoughts Not Your Own | Felt as if the thoughts in your head were not your own? |
|  | Third Party Auditory Hallucinations | Heard voices talking to each other when you were alone? |
|  | Heard Voices (when alone) | Heard voices when you were alone? |
|  | Feel Threatened by Others | Felt that many people around you might hurt or harm you in some way? |
|  | People Are Against You | Felt as if many people around you were plotting against you? |
|  | Thought Withdrawal | Felt as if the thoughts in your head are being taken away from you? |
|  | | |
|  |  | *Have you ever experienced a definite period where for more than two (2) or three (3) days:* |
| **Hypo-manic-like** | Feel Elated | You felt much happier or more cheerful than usual? |
|  | Increased Self-Esteem/Self-Confidence | You felt much more self-confident than usual? |
|  | Impaired Sleep (Reduced Sleep Need) | You needed much less sleep than usual? |
|  | Increased Psychomotor Speed (Speech) | You talked much more than usual? |
|  | Increased Activity (Physical) | You were much more active than usual? |

**Supplementary Table 2. Numbers and proportions of participants endorsing symptoms (N=1904).**

| **Total number**  **of symptoms** | **N (%*)** |
| --- | --- |
| 0 | 293 (15.4) |
| 1 | 145 (7.6) |
| 2 | 164 (8.6) |
| 3 | 141 (7.4) |
| 4 | 171 (9.0) |
| 5 | 147 (7.7) |
| 6 | 143 (7.5) |
| 7 | 141 (7.4) |
| 8 | 99 (5.2) |
| 9 | 98 (5.1) |
| 10 | 89 (4.7) |
| 11 | 60 (3.2) |
| 12 | 61 (3.2) |
| 13 | 46 (2.4) |
| 14 | 31 (1.6) |
| 15 | 29 (1.5) |
| 16 | 19 (1.0) |
| 17 | 12 (0.6) |
| 18 | 5 (0.3) |
| 19 | 4 (0.2) |
| 20 | 3 (0.2) |
| 21 | 1 (0.0) |
| 22 | 2 (0.1) |
| 23 | 0 (0.0) |

Note: * Rounded to the nearest decimal place

**Supplementary Table 3. Relationships between individual symptom items and “days out of role” in a community sample of young adults.**

|  | **APR**  **Whole sample** ^†^**^,^** ^‡^**^,^** ^§^ | **Raw**  **P-value** | **FDR-adjusted**  **P-value** |
| --- | --- | --- | --- |
| ***Symptom item*** |  |  |  |
| Nervous/Tense | 1.12 (0.87 – 1.44) | NS | NS |
| Unhappy/Depressed | 1.25 (0.96 – 1.62) | NS | NS |
| Feel Stressed | 1.03 (0.80 – 1.33) | NS | NS |
| Feel Overwhelmed | 1.27 (0.98 – 1.65) | NS | NS |
| Lost Confidence | 1.04 (0.78 – 1.37) | NS | NS |
| Hopelessness | 1.36 (1.01 – 1.83) | * | NS |
| Somatic pain | 1.01 (0.75 – 1.34) | NS | NS |
| Hypersomnia | 1.27 (1.02 – 1.59) | * | NS |
| Fatigue | 1.34 (1.06 – 1.69) | * | * |
| Poor Sleep (Quality) | 1.20 (0.97 – 1.48) | NS | NS |
| Impaired Concentration | 1.59 (1.26 – 2.00) | *** | *** |
| Anergia | 1.20 (0.91 – 1.58) | NS | NS |
| Feeling Elated | 1.02 (0.76 – 1.36) | NS | NS |
| Increased Self-Confidence/Self-Esteem | 0.95 (0.70 – 1.28) | NS | NS |
| Need Less Sleep | 1.01 (0.79 – 1.29) | NS | NS |
| Increased Psychomotor Speed (Speech) | 1.40 (1.09 – 1.80) | * | * |
| Increased Activity (Physical) | 1.12 (0.87 – 1.45) | NS | NS |
| Thoughts Not Your Own | 1.20 (0.84 – 1.72) | NS | NS |
| Third Party Auditory Hallucinations | 1.75 (0.81 – 3.79) | NS | NS |
| Heard Voices (When Alone) | 0.95 (0.56 – 1.63) | NS | NS |
| Feeling Threatened by Others | 1.42 (0.91 – 2.22) | NS | NS |
| Thinking People are Against You | 0.84 (0.54 – 1.32) | NS | NS |
| Thought Withdrawal | 1.04 (0.47 – 2.30) | NS | NS |
| ***Age*** | 0.99 (0.97 – 1.02) | NS | NS |
| ***Sex*** |  |  |  |
| Female | 1.00 |  |  |
| Male | 0.67 (0.54 – 0.83) | *** | *** |
| ***Twin status*** |  |  |  |
| Not a twin | 1.00 |  |  |
| Monozygotic | 1.21 (0.94 – 1.55) | NS | NS |
| Dizygotic | 1.17 (0.92 – 1.48) | NS | NS |

**Note**:

APR=Adjusted prevalence ratio; ^†^ Adjusted for age; ^‡^ Adjusted for zygosity; ^§^ Adjusted for sex.

*p<0.05

**p<0.01

***p<0.001

NS=non-significant (P>0.05)

**Supplementary Table 4. Numbers and proportions of participants endorsing psychotic-like symptoms (N=1904).**

| **Total number of**  **psychotic-like symptoms** | **N (%*)** |
| --- | --- |
| 0 | 1618 (85.0) |
| 1 | 189 (9.9) |
| 2 | 55 (2.9) |
| 3 | 23 (1.2) |
| 4 | 10 (0.5) |
| 5 | 4 (0.2) |
| 6 | 5 (0.3) |
